# Olive Oil Consumption and Age-Related Macular Degeneration: The Alienor Study

**DOI:** 10.1371/journal.pone.0160240

**Published:** 2016-07-28

**Authors:** Audrey Cougnard-Grégoire, Bénédicte M. J. Merle, Jean-François Korobelnik, Marie-Bénédicte Rougier, Marie-Noëlle Delyfer, Mélanie Le Goff, Cécilia Samieri, Jean-François Dartigues, Cécile Delcourt

**Affiliations:** 1 Univ Bordeaux, ISPED, F-33000 Bordeaux, France; 2 Inserm U1219 –Bordeaux Population Health Research Center, F-33000 Bordeaux, France; 3 CHU de Bordeaux, Service d’Ophtalmologie, Bordeaux, F-33000, France; Tufts University, UNITED STATES

## Abstract

**Background:**

Olive oil provides a mixture of lipids and antioxidant nutrients which may help preventing age-related diseases such as age-related macular degeneration (AMD). However, little is known about the associations between olive oil consumption and the risk of AMD.

**Objective:**

To examine associations between olive oil use and AMD prevalence in elderly subjects.

**Methods:**

Alienor (Antioxydants, Lipides Essentiels, Nutrition et maladies OculaiRes) is a population-based study on eye diseases performed in elderly residents of Bordeaux (France). In 1999–2000, frequencies of consumption of main categories of dietary fats used were collected. In 2006–2088, AMD was graded from non mydriatic retinal photographs into three exclusive stages: no AMD, early AMD, and late AMD. Two categories of preferred dietary fat used (olive oil, n-3 rich oils, n-6 rich oils, mixed oils, butter and margarine) were defined: “no use” and “regular use” (using fat for spreading and/or cooking and/or dressing). Associations of AMD with each fat use were estimated using Generalized Estimating Equation logistic regressions models.

**Results:**

Our study included 654 subjects (1269 eyes) with complete data (n = 268 eyes with early AMD and n = 56 with late AMD). After adjustment for potential confounders, regular use of olive oil was significantly associated with a decreased risk of late AMD (odds ratio [OR] = 0.44, 95% confidence interval [CI]: 0.21;0.91). In contrast, regular use of olive oil was not significantly associated with early AMD (OR = 0.84, 95%CI: 0.59;1.21). No associations were found between regular consumption of n-3 rich oils, n-6 rich oils, mixed oils, butter and margarine and AMD, whatever the stage.

**Conclusions:**

This study suggests a protective effect of olive oil consumption for late AMD in this elderly community-dwelling population. Characterization of the mediating nutrients deserves further research.

## Introduction

Age-related macular degeneration (AMD) is the leading cause of visual impairment among older adults in Europe and in the United States [1, 2]. Worldwide, the number of people with AMD is projected to increase by approximatively 40% from 2020 to 2040 [3]. AMD is a multifactorial disease including genetic and environmental factors, mainly smoking and nutrition.[4] Diet is emerging as a potentially important modifiable risk factor and could be a great value to reduce AMD burden. Epidemiological studies have suggested that nutritional factors and dietary patterns may influence onset and progression of AMD [5–10].

Greater adherence to the Mediterranean diet (MD) has been linked to a reduced risk of overall mortality, and a lower incidence on chronic diseases such as cardiovascular diseases (CVD), neurodegenerative diseases [11–13], cancer [14] and a lower risk of AMD [15, 16]. Health benefits of the MD have been attributed in part to the high consumption of olive oil, a key component of the MD. [17]. Besides its content in mono unsaturated fatty acids (MUFAs), olive oil is a rich source of polyphenols which have been reported to have antioxidant, anti-inflammatory, antithrombotic and anti-microbial properties [17, 18]. Olive oil consumption was inversely associated with CVD and mortality [19–21] and with a lower risk of stroke [22, 23]. The PREDIMED study, a randomized controlled trial recently showed that a MD enriched with virgin olive oil, specifically extra-virgin oil, reduced by 35% (total olive oil) to 39% (extra virgin oil) the risk of suffering of CVD, and by 48% the risk of cardiovascular mortality, in high-risk subjects, in comparison with a low fat diet [21].

However, literature on olive oil and eye diseases is scarce. An in vitro study has reported that hydroxytyrosol, a polyphenol of olive oil, may prevent the degeneration of retinal pigment epithelial cells induced by oxidative stress [24]. In an Australian cohort [25] of 6,734 individuals aged 58 to 69, olive oil intake (≥ 100 mL/week vs. <1 mL/week) was significantly associated with a lower prevalence of late AMD.

Although many epidemiological studies have been conducted on the potential associations of AMD with nutrition, little is known about the associations between olive oil consumption and AMD, independently of other lifestyles factors. Thus our objective is to examine associations between olive oil use and AMD prevalence in a population-based study of French elderly subjects.

## Subjects and Methods

### Study Purpose

The Alienor (Antioxydants, LIpides Essentiels, Nutrition et maladies OculaiRes) study is a population-based study aimed at assessing the associations of nutritional factors (in particular antioxidants, macular pigment, and fatty acids), determined from plasma measurements and estimations of dietary intake with age-related eye diseases (AMD, glaucoma, cataract, dry eye syndrome) [26]. It also takes into account other major determinants of eye disease, including genetic polymorphisms, lifestyle and vascular factors.

### Study Sample

Subjects of the Alienor study were recruited from an ongoing population-based study (Three-City [3C] study) on the vascular risk factors for dementia [27], as previously described. The 3C study included 9294 community-dwelling persons aged 65 years and older from three French cities (Bordeaux, Dijon and Montpellier), among whom 2104 were recruited in Bordeaux. Subjects were initially recruited in 1999 to 2001 and were followed-up about every 2 years since baseline. At cohort baseline, a standardized questionnaire was administered including a broad food frequency questionnaire. Data collected at each examination included cognitive testing with diagnoses of dementia and assessment of vascular risk factors. In addition, fasting blood and DNA samples were collected at baseline and kept frozen at -80°C.

The Alienor study consisted in an eye examination, which was proposed to all participants of the third follow-up (2006–2008) of the 3C cohort in Bordeaux. Among the 1450 participants re-examined in 2006 to 2008, 963 (66.4%) participated in the Alienor study. Detailed characteristics of participants and nonparticipants have been described elsewhere [26].

This research followed the tenets of the Declaration of Helsinki. Participants gave written consent for participation in the study. The design of the Alienor study has been approved by the Ethical Committee of Bordeaux (Comité de Protection des Personnes Sud-Ouest et Outre-Mer III) in May 2006.

### Eye Examination

The eye examination took place in the Department of Ophthalmology of the University Hospital of Bordeaux, France. It included a recording of ophthalmological history, measures of visual acuity, refraction, two 45°nonmydriatic color retinal photographs (one centered on the macula, the other centered on the optic disc), measures of intraocular pressure and central corneal thickness, and break-up time test.

Retinal photographs were performed using a high-resolution digital non-mydriatic retinograph (TRC NW6S; Topcon, Tokyo, Japan). Photographs were interpreted in duplicate by two specially trained technicians. Inconsistencies between the two interpretations were adjudicated by a retina specialist for classification of AMD and other retinal diseases and by a glaucoma specialist for classification of glaucoma. All cases of late AMD, other retinal diseases, and glaucoma were reviewed and confirmed by specialists.

### Classification of AMD

Retinal photographs were interpreted according to the international classification [28] and to a modification of the grading scheme used in the Multi-ethnic Study of Atherosclerosis (MESA) for drusen size, location, and area [29]. Late AMD was defined by the presence of neovascular AMD or geographic atrophy within the grid (3000 μm from the foveal center). Neovascular AMD included serous or hemorrhagic detachment of the retinal pigment epithelium (RPE) or sensory retina, subretinal or sub-RPE hemorrhages and fibrous scar tissue. Geographic atrophy was defined as a discrete area of retinal depigmentation, 175 μm in diameter or larger, characterized by a sharp border and the presence of visible choroidal vessels. Five cases of late AMD had no gradable photographs and were classified by using ophthalmic history of AMD and AMD therapy (in particular antiangiogenic agents and photodynamic therapy), and confirmed by their treating ophthalmologist.

Early AMD was defined by the presence of soft distinct drusen and/or soft indistinct drusen and/or reticular drusen and/or pigmentary abnormalities, in the absence of late AMD. Soft distinct and soft indistinct drusen were larger than 125 μm in diameter and with uniform density and sharp edges or decreasing density from the center outward and fuzzy edges, respectively. Pigmentary abnormalities were defined as areas of hyperpigmentation and/or hypopigmentation (without visibility of choroidal vessels).

### Nutritional data

Preferred dietary fats used for dressing, cooking and spreading and frequency of consumption of broad categories of foods were recorded at baseline of the 3C study (1999–2001), through an standardized brief 30-item food frequency questionnaire, given at home by a trained interviewer, as previously described [30].

Participants were invited to indicate their preferred dietary fats used at least once a week for dressing, cooking or spreading among the following list: butter, margarine, corn oil, peanut oil, sunflower or grape seed oil, olive oil, mixed oil, colza oil, walnut oil and soya oil. Colza, walnut and soya oils were grouped in n-3 rich oils, since they contain more than 5% n-3 fatty acids; peanut, sunflower, grape seed oil and corn oil were grouped in n-6 rich oil, since they contain more than 30% n-6 fatty acids and less than 1% n-3 fatty acids. Frequency of preferred dietary fat used was divided into two groups of users: “non-users” and “regular users” (using fat for spreading and/or dressing and/or cooking).

Regarding consumption of foods, the questionnaire included 9 broad food categories: (1) meat and poultry, (2) fish (including seafood), (3) eggs, (4) milk and dairy products, (5) cereals, (6) raw fruits, (7) raw vegetables, (8) cooked fruits and vegetables and (9) legumes. Frequency of consumption food was recorded in six modalities: “never, less than once a week, once a week, 2–3 times a week, 4–6 times a week, and daily”. Quantities of wine, beer and other alcohols (aperitifs or after-dinner drinks) were estimated. Frequency of food consumption was divided into two groups of consumers: “occasional” and “regular”. Occasional consumers were defined as individuals who declared eating a given food infrequently (below the 10^th^ percentile of the distribution). Dietary habits and frequency of food consumption of the 3C cohort have been described elsewhere [31].

Mean total energy intake per day was estimated from a 24-hour dietary recall performed during a face-to-face interview administered by trained dieticians in 2001–2002. [32].

### Other Variables

Data were collected during a face-to-face interview using a standardized questionnaire administered by a trained psychologist or nurse in 1999–2001. Demographic variables included age, gender, educational level and monthly income. Medical variables included cardiovascular disease (self-reported history of cardiovascular disease including stroke, angina pectoris, myocardial infarction, and cardiac and vascular surgery), diabetes (self-reported or fasting blood glucose ≥7.0 mmol/L or nonfasting blood glucose ≥11.0 mmol/L or antidiabetic medication), hypertension (systolic blood pressure≥140mmHg or diastolic blood pressure≥ 90mmHg or antihypertensive treatment), body mass index (BMI, [weight (kg)/ height^2^ (m^2^)]) and an inventory of all drugs used during the preceding month. Medical prescriptions and, where feasible, the medications themselves were seen by the interviewer. The name of the medication was recorded, and all drugs were subsequently coded according to the World Health Organization (WHO) anatomical therapeutic chemical (ATC) classification [33].

Plasma fatty acid composition was determined from the fasting blood samples collected at the baseline examination of the 3C study (1999–2001) after storage at -80°C for 36 months [34–36]. No participant declared using oral n-3 fatty acid supplementation. The results of each fatty acid were expressed as the percentage of total fatty acids.

Biological data were collected at the same time as the blood collection for plasma fatty acid measurements and included plasma lipids (total cholesterol (TCH), low-density lipoprotein cholesterol (LDL), high-density lipoprotein cholesterol (HDL), and triglycerides (TG)) and genetic factors (*CFH* rs1061170 and *ARMS2* rs10490924, *LPL* rs12678919 and *LIPC* rs493258), which have been shown to be very strong predictors of risk for AMD [37–39], including in the Alienor study [40–42]. Plasma lipids were measured at the Biochemistry Laboratory of the University Hospital of Dijon from baseline fasting blood samples by using routine enzymatic techniques and genetic polymorphisms were determined by the Lille Génopôle, from the DNA samples.

Lifestyle variables included smoking in pack-years [pack-years = number of packs (of 20 cigarettes) smoked per day × number of years of smoking) and physical activity which was assessed by two questions: “Do you practice sports?” and “Do you perspire when you practice sports?” A three-level variable was computed to describe the intensity of physical activity: none, moderate, or high intensity [32].

### Data analysis

Results were presented using means ± standard deviations (SDs) for continuous variables, and *n* and percentage for non-continuous variables.

We first compared characteristics of subjects with and without missing data. For continuous and categorical variables the comparisons were assessed using logistic regression adjusted for age.

Associations of baseline demographic, behavioral, medical, genetic and dietary characteristics with olive oil consumption were examined with Student t-test and Chi-square test, as appropriate.

Associations between olive oil consumption and other preferred fats use and of early AMD and late AMD prevalences with were estimated using logistic generalized estimating equations (GEE) models taking into account data from both eyes and their intra-individual correlations [43]. The associations are presented as odds ratios (ORs) and 95% confidence intervals (CIs). Eyes without AMD were the reference in all models (early and late AMD).

Analyses were first adjusted for variables significantly associated with olive oil consumption in the univariate analyses (p-value < 0.05), and for major risk factors related to AMD in our study (smoking, plasma HDL-cholesterol, plasma total n-3 polyunsaturated fatty acids (PUFAs)) (model 1) [35, 40, 44]. We performed further adjustments for major genetic risk factors for AMD in our study (*CFH* rs1061170, *ARMS2* rs10490924, *LPL* rs12678919 and *LIPC* rs493258 polymorphisms) (model 2) [40–42]. In addition, age, sex, BMI and total energy intake were forced into all multivariable analyses.

Potential interactions between olive oil consumption and genetic polymorphisms were assessed. Genetic polymorphisms were introduced in the models one by one. We withdrew interaction terms when not statistically significant (*P global*>0.05).

P-values<0.05 were considered as significant. All statistical analyses were performed using SAS version 9.3 (SAS Institute Inc, Cary, NC; procedure GENMOD for the GEE analysis).

## Results

Among the 963 subjects of the Alienor study, olive oil consumption was available in 959 subjects (99.6%). Of those, 874 subjects (1690 eyes) had complete data for AMD status in at least one eye. Among them, 654 (1269 eyes) had complete data for potential confounders, and were used for the estimation of associations of AMD with olive oil consumption. Among them, 189 (268 eyes) had early AMD and 36 (56 eyes) had late AMD.

Participants with complete data (n = 654) were younger than those with missing data (n = 309) (p = 0.007). After adjustment for age, they were also more frequently married (p = 0.008), did less often physical activity (p<0.0001), presented less often diabetes (p = 0.04), had a lower mean level of plasma triglycerides (p = 0.02), used more frequently olive oil (p = 0.02), margarine (p = 0.001), and consumed more often raw vegetables (p = 0.03). Participants with complete data did not differ significantly from those with missing data for history of cardiovascular disease, smoking, hypertension, BMI, plasma TCH, HDL, oleic acid, n-3 PUFAs, n-6 PUFAs, saturated fatty acids, and *CFH* rs1061170, *ARMS2* rs10490924, *LPL* rs12678919,*LIPC* rs493258 polymorphisms, and regular consumption of fish, meat, raw fruits, cooked fruits, vegetables, legumes, dairy products, eggs, and regular use of n-3, n-6 rich oils and butter (**S1 Table**).

The final sample consisted of 654 individuals, 253 men (38.7%) and 401 women (61.3%), aged 72.7 years (range: 65.6–87.1) on average at baseline in 1999–2001. As shown in **Table 1,** regular users of olive oil were more educated, were more frequently married, and with borderline significance had a higher monthly income than non-users. No significant associations were found between olive oil use and age, gender, smoking, physical activity or alcohol use.

**Table 1:** Baseline sociodemographic characteristics and lifestyle factors of the 654 participants of the Alienor study (1999–2001), according to olive oil use^1^.

		Olive oil use		
		Non user	Regular user	P-value^2^
		(N = 175)	(N = 479)	
Gender, Women		105 (60.0)	296 (61.8)	0.68
Age (years)		73.5 ± 4.2	72.8 ± 4.4	0.51
Education				
	None or primary	63 (36.0)	117 (24.4)	0.004
	Secondary	50 (28.6)	131 (27.4)	
	High school or university	62 (35.4)	231 (48.2)	
Monthly income (in euros)				
	<1500	75 (42.9)	164 (34.2)	0.05
	[1500–2250[	38 (21.7)	140 (29.2)	
	≥2250	51 (29.1)	157 (32.8)	
	Refused to answer	11 (6.3)	18 (3.8)	
Marital status				
	Married	98 (56.0)	317 (66.2)	0.02
	Divorced, widowed or single	77 (44.0)	162 (33.8)	
Smoking (pack-years)				
	No	113 (64.6)	310 (64.7)	0.25
	<20	25 (14.3)	89 (18.6)	
	≥20	37 (21.1)	80 (16.7)	
Alcohol use (number of glasses per week)		11.5 ± 13.5	10.4 ± 11.7	0.33
Physical activity				
	None	104 (59.4)	259 (54.1)	0.36
	Medium	30 (17.1)	109 (22.8)	
	High	17 (9.7)	54 (11.3)	
	Not answered	24 (13.7)	57 (11.9)	

ALIENOR: Antioxydants, Lipides Essentiels, Nutrition et maladies OculaiRes

^1^ Values are means ± SDs or n (%).

^2^ Chi-square test for categorical variables and Student test for continuous variables.

Regarding health indicators, plasma lipids and PUFAs measurements (**Table 2**), regular users of olive oil presented a higher mean level of plasma oleic acid and a lower mean level of plasma n-6 PUFAs. There were no statistically significant associations of regular olive oil use with hypertension, systolic and diastolic blood pressures, antihypertensive therapy, hypercholesterolemia, history of cardiovascular disease, diabetes, BMI, plasma total, LDL, HDL-cholesterol or triglycerides nor with plasma n-3 PUFAs or saturated fatty acids.

**Table 2:** Baseline health indicators, plasma lipids and fatty acids measurements of the 654 participants of the Alienor study (1999–2001) according to olive oil use^1^.

	Olive oil use		
	Non user	Regular user	P-value^2^
	(N = 175)	(N = 479)	
Hypertension^3^	135 (77.1)	351 (73.3)	0.32
SBP (mmHg)	149.9 ± 19.8	142.4 ± 20.3	0.15
DBP (mmHg)	82.5 ± 11.2	81.0 ± 10.3	0.11
Antihypertensive therapy	85 (48.6)	246 (51.4)	0.53
Diabetes^4^	18 (10.3)	30 (6.3)	0.08
Hypercholesterolemia	87 (49.7)	255 (53.2)	0.42
History of cardiovascular disease	14 (8.0)	39 (8.1)	0.95
BMI	26.6 ± 4.0	26.2 ± 3.7	0.30
Plasma triglycerides, (mmol/l)	1.28 ± 0.7	1.28 ± 0.6	0.07
Plasma total cholesterol, (mmol/l)	5.79 ± 1.0	5.74 ± 1.0	0.56
Plasma LDL-cholesterol, (mmol/l)	3.61 ± 0.8	3.61 ± 0.8	0.99
Plasma HDL-cholesterol, (mmol/l)	1.61 ± 0.4	1.60 ± 0.4	0.91
Plasma Oleic acid^5^	20.1 ± 3.2	20.9 ± 3.3	0.006
Plasma n-3 PUFAs^5^	4.3 ± 1.2	4.5 ± 1.3	0.06
Plasma n-6 PUFAs^5^	33.7 ± 5.1	32.7 ± 4.9	0.02
Plasma saturated fatty acids^5^	39.6 ± 4.6	39.7 ± 5.8	0.91

ALIENOR: Antioxydants, Lipides Essentiels, Nutrition et maladies OculaiRes; BMI: body mass index; SBP: systolic blood pressure; BDP: diastolic blood pressure; HDL: High-density lipoprotein cholesterol; LDL: Low-density lipoprotein cholesterol; PUFAs: polyunsaturated fatty acids

^1^ Values are means ± SDs or n (%)

^2^ Chi-square test for categorical variables and Student test for continuous variables

^3^ Average systolic blood pressure ≥ 140 mmHg and/or average diastolic blood pressure ≥ 90 mmHg and/or antihypertensive medication use

^4^ Fasting blood glucose ≥ 7 mmol/L and/or nonfasting blood glucose ≥11.0 mmol/L and/or antidiabetic medication use

^5^ Percentages of total fatty acids

Concerning baseline nutritional factors and preferred dietary fats used (**Table 3**), regular users of olive oil consumed more regularly raw fruits, cooked fruits and vegetables, used less preferentially n-6 rich oils, and with borderline significance they consumed more regularly dairy products. No significant associations were found between regular olive oil use and regular consumption of fish, meat, raw vegetables, pulses, eggs and with preferred fats of mixed oil, n-3 rich oils, butter and margarine.

**Table 3:** Baseline nutritional factors and preferred dietary fats used of the 654 participants of the Alienor study (1999–2001) according to olive oil use^1^.

	Olive oil use		
	Non user	Regular user	P-value^2^
	(N = 175)	(N = 479)	
**Regular consumption of**			
Fish (≥ once a week)	157 (89.7)	440 (91.9)	0.39
Meat (≥ twice a week)	166 (94.9)	457 (95.4)	0.77
Raw vegetables (≥ twice a week)	156 (89.1)	442 (92.3)	0.21
Raw fruits (≥ 4 times a week)	140 (80.0)	420 (87.7)	0.01
Cooked fruits and vegetables (≥ 4 times a week)	146 (83.4)	436 (91.0)	0.006
Legumes (≥ once a week)	157 (89.7)	441 (92.1)	0.34
Dairy products (once a day)	161 (92.0)	458 (95.6)	0.07
Eggs (once a week)	141 (80.0)	390 (81.4)	0.81
**Preferred fats use**			
N-3 rich oils^3^	11 (6.3)	36 (7.5)	0.59
N-6 rich oils^4^	146 (83.4)	302 (63.1)	<0.0001
Mixed oil	34 (19.4)	94 (19.6)	0.96
Butter	100 (57.1)	284 (59.3)	0.62
Margarine	54 (30.9)	148 (30.9)	0.21

ALIENOR: Antioxydants, Lipides Essentiels, Nutrition et maladies OculaiRes

^**1**^ Values are n (%)

^2^ Chi-square test

^3^ colza, walnut or soya oils

^4^ peanut, sunflower, grape seed or corn oils

Regarding genetic characteristics (**Table 4**), there were no statistically significant associations of regular olive oil use with *CFH* rs1061170, *ARMS2* rs10490924, *LPL* rs12678919 or *LIPC* rs493258 polymorphisms.

**Table 4:** Baseline genetic characteristics of the 654 participants of the Alienor study (1999–2001) according to olive oil use^1^.

	Olive oil use		
	Non user	Regular user	P-value^2^
	(N = 175)	(N = 479)	
*CFH* rs1061170 (n = 634)			
TT (low AMD risk)	80 (47.9)	211 (45.2)	0.23
TC	64 (38.3)	209 (44.8)	
CC (high AMD risk)	23 (13.8)	47 (10.1)	
*ARMS2* rs10490924 (n = 583)			
GG (low AMD risk)	99 (63.1)	274 (64.3)	0.95
GT	51 (32.5)	135 (31.7)	
TT (high AMD risk)	7 (4.5)	17 (4.0)	
*LPL* rs12678919 (n = 567)			
A A (low AMD risk)	107 (70.4)	309 (74.5)	0.39
AG	40 (26.3)	99 (23.9)	
GG (high AMD risk)	5 (13.3)	7 (1.7)	
*LIPC* rs493258 (n = 583)			
CC (high AMD risk)	41 (26.1)	125 (29.3)	0.70
CT	77 (49.0)	205 (48.1)	
TT (low AMD risk)	39 (24.8)	96 (22.5)	

ALIENOR: Antioxydants, Lipides Essentiels, Nutrition et maladies OculaiRes

^**1**^ Values are n (%)

^2^ Chi-square test

Regarding the other potential dietary fats used (S2 and S3 Tables), regular users of n-3 rich oils consumed alcohol and eggs more frequently and more often presented with a history of cardiovascular disease than non-users of n-3 rich oils. Regular users of n-6 rich oils were more likely to have no formal education or a primary school level of education, higher systolic and diastolic blood pressures and a higher mean level of plasma n-6 PUFAs, and lower mean levels of plasma oleic acid and n-3 PUFAs than non-users of n-6 rich oils. Regular users of mixed oils, had lower mean plasma total and HDL-cholesterol concentrations and a higher frequency of the ‘A A’ (low AMD risk) allele of the LPL gene, and consumed raw fruits more frequently than non-users of mixed oils. Regular users of butter were more likely to have high diastolic blood pressure, and a low BMI and consumed eggs more frequently than non-users of butter. Finally, regular users of margarine reported less physical activity, more frequent consumption of cooker fruits and vegetables and had higher mean n-6 PUFAs levels than non-users of margarine.

**Table 5** displays the associations of AMD with regular consumption of olive oil, n-3 rich oils, n-6 rich oils, mixed oils, butter and margarine. After multivariate adjustment (model 1), regular consumption of olive oil was significantly associated with late AMD (OR = 0.44, 95%CI: 0.21;0.91, p = 0.03), but not with early AMD (OR = 0.84, 95%CI: 0.59;1.24, p = 0.36). No associations were found between regular consumption of n-3 rich oils, n-6 rich oils, mixed oils, butter and margarine and AMD, whatever the stage.

**Table 5:** Associations of preferred dietary fats used with AMD^1^ in the Alienor study (odds-ratios (OR) and [95% confidence interval (CI)]) (N = 654 subjects, n = 1269 eyes).

	No AMD	Early AMD		Late AMD	
	(n = 945 eyes)	(n = 268 eyes)		(n = 56 eyes)	
	N (%)	N (%)	OR	N (%)	OR
			[95%CI]		[95%CI]
**Olive Oil**					
No	233 (24.7)	77 (28.7)	1.0 (ref)	23 (41.1)	1.0 (ref)
Yes	712 (75.3)	191 (71.3)	0.84	33 (58.9)	0.44
			[0.59;1.21]		[0.21;0.91]
*P value*			*0*.*36*		*0*.*03*
**n-3 rich oils**^2^					
No	877 (92.8)	246 (91.8)	1.0 (ref)	55 (98.2)	1.0 (ref)
Yes	68 (7.2)	22 (8.2)	1.19	1 (1.8)	0.35
			[0.67;2.11]		[0.06;2.08]
*P value*			*0*.*54*		*0*.*25*
**n-6 rich oils**^3^					
No	299 (31.6)	86 (32.1)	1.0 (ref)	20 (35.7)	1.0 (ref)
Yes	646 (68.4)	182 (67.9)	0.98	36 (64.3)	0.88
			[0.68;1.40]		[0.42;1.87]
*P value*			*0*.*91*		*0*.*74*
**Mixed oils**					
No	764 (80.8)	216 (80.6)	1.0 (ref)	39 (69.6)	1.0 (ref)
Yes	181 (19.2)	52 (19.4)	1.09	17 (30.4)	1.96
			[0.70;1.68]		[0.83;4.59]
*P value*			*0*.*71*		*0*.*12*
**Butter**					
No	394 (41.7)	108 (40.3)	1.0 (ref)	25 (44.6)	1.0 (ref)
Yes	551 (58.3)	160 (59.7)	0.94	31 (55.4)	0.75
			[0.67;1.32]		[0.35;1.59]
*P value*			*0*.*73*		*0*.*45*
**Margarine**					
No	658 (69.6)	179 (66.8)	1.0 (ref)	41 (73.2)	1.0 (ref)
Yes	287 (30.4)	89 (33.2)	1.12	15 (26.8)	1.02
			[0.79;1.61]		[0.45;2.29]
*P value*			*0*.*53*		*0*.*97*

ALIENOR: Antioxydants, Lipides Essentiels, Nutrition et maladies OculaiRes; AMD: age-related macular degeneration; GEE: generalized estimating equations; PUFAs: polyunsaturated fatty acids

^1^ Logistic GEE separate models adjusted for age, gender, educational level, marital status, smoking, BMI, regular consumption of raw fruits, regular consumption of cooked fruits and vegetables, plasma HDL-cholesterol, plasma total n-3 PUFAs, plasma total n-6 PUFAs and total energy intake. Eyes without AMD were the reference in all models (early and late AMD)

^2^ colza, walnut or soya oils

^3^ peanut, sunflower, grape seed or corn oils

Among subjects with available genetic data (N = 1067 eyes), associations between consumption of olive oil and late AMD were even stronger after further adjustments for genetic factors (OR = 0.27, 95%CI: 0.11;0.65, p = 0.003 for late AMD and OR = 0.92, 95%CI: 0.61;1.38, p = 0.69 for early AMD). As with model 1, no associations were found between regular consumption of n-3 rich oils, n-6 rich oils, mixed oils, butter and margarine when we further adjusted for genetic factors and any stages of AMD (data not shown).

Finally, we detected no significant interaction between genetic factors (*CFH* rs1061170, *ARMS2* rs10490924, *LPL* rs12678919 and *LIPC* rs493258 polymorphisms) and consumption of olive oil (all p>0.05) for early AMD models and no interactions for late AMD models (except for LIPC and LPL in late AMD models, due to insufficient power, the test of interactions for those two genes were non applicable).

## Discussion

This study reported a decreased risk of late AMD among olive oil users, after adjustment for multiple potential confounders. Association between olive oil use and early AMD was not significant. The consumption of other types of fats was not associated with any stages of AMD.

In the literature, few studies have explored the association between different types of oils or fats use with AMD. They have mainly examined the associations of early and late AMD with dietary fatty acids (MUFAs, saturated and PUFAs or trans-fat) [9, 10, 15, 45–48]. Indeed, interest in dietary fat and AMD has been primarily centred on saturated and PUFAs or trans-fat rather than on type of cooking oils and fats. In addition, the studies which have explored the associations between dietary fat and AMD are mainly from North America [15, 45–48] where consumption of olive oil is not frequent.

To our knowledge, only one published study has examined the associations between olive oil use and AMD [25]. In this Australian cohort of 6734 persons aged aged 63.7 year on average at baseline, Chong et al.[25] reported an inverse association between a higher intake of olive oil (≥100 mL/week vs <1 mL/week) and the prevalence of late AMD, after adjustments for age, smoking, energy, vitamins C and E, beta carotene, zinc, lutein, zeaxanthin and supplements (OR = 0.48, 95%CI: 0.22;1.04). The magnitude of the association reported between olive oil and late AMD in our study (OR = 0.44, 95%CI: 0.21;0.91) is strikingly similar to theirs. As in the present study, they did not report any association with early AMD (OR: 1.00, 95%CI: 0.81;1.24). The two studies present similar design. The participants of the Australian and French cohort study were selected from ongoing cohorts, the Melbourne Collaborative Cohort Study, and the 3C-study, respectively. The participants of the two studies were residents from two big cities (Melbourne and Bordeaux) and were recruited via electoral registers. The distribution of females were 61% for both studies and the dietary intake were collected a decade before the ophthalmological examination. However the prevalence of late AMD was 3 fold higher in the Alienor study in comparison with the Melbourne study explained in part by the older age of the French participants in comparison with the Australians (10 years older in average). In their study, dietary intake from the year before baseline was estimated using food frequency questionnaires with 121-items allowing them to estimate the quantities of olive oil consumed. Thus, findings are not directly comparable as we did not assess the quantities of olive oil consumed in our study, and the average intake in the “regular user” category certainly differs across the two populations. However, 53.6% of their population consumed at least 1mL/week of olive oil, while in our study 73.2% were considered regular users of olive oil, suggesting that our population from the South of France consumes more olive oil than the Australians.

Olive oil contains 85% of MUFA in the form of oleic acid and other nonfat components of olive oil rich in polyphenols which may contribute to this apparent protective effect. The associations between MUFAs intake and AMD have been inconsistent in the litterature. Some studies reported an increased risk of AMD with high dietary intake of MUFAs [47–49] whereas other reported a decreased risk [50, 51] or no association [10, 46].

Thus, the protective mechanisms underlying the association between olive oil intake and lower odds of late AMD found in our study might rely on minor components with biological properties contained in olive oil, from 1 to 2% of its total content. These minor components are classified into two types, the unsaponifiable and the soluble fraction. The last type includes the phenolic compounds (such as oleuropein, hydroxytyrosol, tyrosol and oleocanthal) which have been reported to have antioxidant and anti-inflammatory properties [17].

The main anti-inflammatory effects seem to be mediated by oleocanthal, which mimics ibuprofen, a non-steroidal anti-inflammatory drug [52]. It has been estimated that the amount of oleocanthal in about 4 tablespoons (50 ml) of extra virgin olive oil works has 10% of the adult ibuprofen dosage for pain relief [52]. In addition, olive oil is also rich in hydroxytyrosol and oleuropein that have natural antioxidant activities [53]. These compounds have been reported to prevent the LDL-cholesterol from oxidation [54] and to reduce the expression of genes and proteins that mediate inflammation [55]. In vitro, hydroxytyrosol has been reported to have a protective effect against oxidative damage in retinal pigment epithelial cells and mitochondrial dysfunction [24]. Hence, it is conceivable that low, continuous doses of a naturally non-steroidal anti-inflammatory drug such as oleocanthal may decrease inflammation over time, as well as reduce oxidative stress with its natural antioxidant compounds and then contribute to reduce the development of chronic inflammatory disease such as AMD. However, at this stage, the mechanism by which olive oil compounds exert their beneficial effects on late AMD is limited and further studies are needed.

Regarding the other fats preferentially used, we did not find any association between margarine, butter use and early or late AMD. Our results are similar to the findings of Chong et al. [25] who did not report association with higher use of margarine and early or late AMD (≥17.5 times/week vs 0 times/week, OR:1.03, 95%CI: 0.83;1.29; OR:1.05, 95%CI: 0.53;2.07, respectively) or use of butter with early and late AMD (≥7 times/week vs 0 times/week, OR:1.06, 95%CI: 0.88;1.29; OR: 0.85, 95%CI: 0.45;1.58, respectively). In addition, we did not report any association between oils rich in n-6 PUFAs and any stages of AMD. Similar results have been found in the study of Zerbib et al. [56] In their large French case-control study (1024 subjects with exudative AMD vs 275 controls), authors did not evidence associations between use of oils rich in n-6 PUFAs and exudative AMD after adjustments for age and gender (OR: 1.23, 95%CI: 0.87;1.74). However, by contrast with our study, they reported a decreased risk of exudative AMD in users of oils rich in n-3 PUFAs, after adjustments for age, gender, smoking, consumption of fruits, waist circumference, CFH and *ARMS2* genetic polymorphisms (OR: 0.55, 95%CI: 0.36;0.84).

Despite the high statistical power in the early AMD group, no associations were found between olive oil and early AMD. This suggests that risk factors may be different at different stages of the disease, some favouring the accumulation of drusen and pigmentary abnormalities, while others may influence more directly neoangiogenesis or apoptosis. For instance, smoking is much more strongly associated with late than early AMD in most studies. Alternatively, early AMD may represent a heterogeneous group of subjects, some of whom actually bear a low risk of developing late AMD, as suggested by some studies [57–60].

A strength of the present study is that major potential confounding factors were taken into account, including socio-demographic status, factors related to nutritional data and to healthy lifestyles such as vegetable and fruits intake, BMI and plasma n-3 PUFA, total energy intake, and the major AMD-related genetic polymorphisms in the Alienor study [40–42]. This may have helped reducing residual confounding and may have minimized the biases. Another strength is that our study was conducted in a population where the intake of olive oil was relatively high, allowing a better assessment of the association between olive oil consumption and AMD.

One limitation of our study is the small number of cases of late AMD, which may have induced insufficient statistical power for detecting some interactions between olive oil and genetic risk particularly those with LIPC and LPL with late AMD. Thus, larger studies are needed to test whether genetic risk can be reduced by dietary nutrients such as olive oil.

Another limitation of our study could come from the representativeness of the sample. As previously discussed [44], the Alienor subsample tends to over-represent younger subjects and high socioeconomic status, compared to the parent cohort (the 3C study) [26]. The individuals included in this study may accordingly be healthier and present different lifestyles, mainly concerning their diet and physical activity, by comparison with the general population. The distribution of the prevalence of eye diseases may have been affected due to these differences. However, the age- and gender-specific prevalence rates of AMD in the Alienor study were similar to those observed in other studies performed in Europe [61, 62] and other industrialized countries [63]. In addition, the analyses were adjusted for dietary intake (fruits and vegetables, plasma n-3 PUFAs, total energy intake), socio-economic status and BMI showing that they have a little impact on the association between olive oil use and late AMD.

Data collection was performed in the same way in all individuals irrespective of their AMD stage and photograph graders had no access to data related to nutritional data, or any other medical or genetic characteristics. Consequently, we can assume that the error was not differential and was unlikely to have biased the estimation of any of the associations of AMD with nutritional data.

Another limitation is that we did not collect the quantity of olive oil use, excluding any quantitative approach. At last, we did not collect the different varieties of olive oil use. It is known that extra virgin oil provides more cardiovascular benefits than refined oil due mainly to its high content in polyphenols. Given that 98% of the French market of olive oil is composed of extra virgin olive oil,[23, 64] we can expect that in our study, olive oil users mostly consumed extra virgin olive oil.

In conclusion, this study suggests that olive oil use is associated with a reduced risk for late AMD in older subjects. These associations require confirmation by other studies and further studies are needed to better understand the potential role of olive oil in AMD.

## Supporting Information

S1 Table Comparison of the characteristics of subjects included with and without missing data (Alienor study 2006–2008, Bordeaux, France)*. ALIENOR: Antioxydants, Lipides Essentiels, Nutrition et maladies OculaiRes; AMD: age-related macular degeneration; BMI: body mass index; HDL: High-density lipoprotein cholesterol; LDL: Low-density lipoprotein cholesterol; PUFA: polyunsaturated fatty acids; S.D.: standard deviation; * Values are means ± SDs or n (%).^†^Comparisons of characteristics with missing data status were examined with logistic regression analysis adjusted for age; ^‖^ Fasting blood glucose ≥ 7 mmol/L and/or nonfasting blood glucose ≥11.0 mmol/L and/or antidiabetic medication use; ^§^ Average systolic blood pressure ≥ 140 mmHg and/or average diastolic blood pressure ≥ 90 mmHg and/or antihypertensive medication use; ^{^percentage of total fatty acids; ^#^ colza, walnut or soya oils; ** peanut, sunflower, grape or corn oils
(DOCX)

S2 Table comparison of the characteristics of subjects with n-3, n-6 rich oils and mixed oils use (Alienor study 2006–2008, Bordeaux, France)*. ALIENOR: Antioxydants, Lipides Essentiels, Nutrition et maladies OculaiRes; S.D.: standard deviation; AMD: age-related macular degeneration; BMI: body mass index; HDL: High-density lipoprotein cholesterol; LDL: Low-density lipoprotein cholesterol; PUFAs: polyunsaturated fatty acids; * Values are means ± SDs or %. ^†^ Chi-square or fisher exact test for categorical variables and Student test for continuous variables; ^§^ Average systolic blood pressure ≥ 140 mmHg and/or average diastolic blood pressure ≥ 90 mmHg and/or antihypertensive medication use; ^‖^ Fasting blood glucose ≥ 7 mmol/L and/or nonfasting blood glucose ≥11.0 mmol/L and/or antidiabetic medication use; ^{^percentage of total fatty acids; ^#^ colza, walnut or soya oils; ** peanut, sunflower, grape or corn oils.
(DOCX)

S3 Table Comparison of the characteristics of subjects with butter or margarine use (Alienor study 2006–2008, Bordeaux, France)*. ALIENOR: Antioxydants, Lipides Essentiels, Nutrition et maladies OculaiRes; S.D.: standard deviation; AMD: age-related macular degeneration; BMI: body mass index; HDL: High-density lipoprotein cholesterol; LDL: Low-density lipoprotein cholesterol; PUFAs: polyunsaturated fatty acids; * Values are means ± SDs or %. ^†^ Chi-square or fisher exact test for categorical variables and Student test for continuous variables;^§^Average systolic blood pressure ≥ 140 mmHg and/or average diastolic blood pressure ≥ 90 mmHg and/or antihypertensive medication use; ^‖^ Fasting blood glucose ≥ 7 mmol/L and/or nonfasting blood glucose ≥11.0 mmol/L and/or antidiabetic medication use; ^{^percentage of total fatty acids; ^#^ colza, walnut or soya oils; ** peanut, sunflower, grape or corn oils.
(DOCX)

## References

[1] Bourne RR, Jonas JB, Flaxman SR, Keeffe J, Leasher J, Naidoo K, et al Prevalence and causes of vision loss in high-income countries and in Eastern and Central Europe: 1990–2010. Br J Ophthalmol. 2014;98(5):629–38. Epub 2014/03/26. 10.1136/bjophthalmol-2013-304033 . 24665132

[2] Bourne RR, Stevens GA, White RA, Smith JL, Flaxman SR, Price H, et al Causes of vision loss worldwide, 1990–2010: a systematic analysis. The Lancet Global health. 2013;1(6):e339–49. Epub 2014/08/12. 10.1016/S2214-109X(13)70113-X . 25104599

[3] Wong WL, Su X, Li X, Cheung CM, Klein R, Cheng CY, et al Global prevalence of age-related macular degeneration and disease burden projection for 2020 and 2040: a systematic review and meta-analysis. The Lancet Global health. 2014;2(2):e106–16. Epub 2014/08/12. 10.1016/S2214-109X(13)70145-1 . 25104651

[4] Lim LS, Mitchell P, Seddon JM, Holz FG, Wong TY. Age-related macular degeneration. Lancet. 2012;379(9827):1728–38. Epub 2012/05/09. 10.1016/S0140-6736(12)60282-7 . 22559899

[5] Amirul Islam FM, Chong EW, Hodge AM, Guymer RH, Aung KZ, Makeyeva GA, et al Dietary patterns and their associations with age-related macular degeneration: the Melbourne collaborative cohort study. Ophthalmology. 2014;121(7):1428–34 e2. Epub 2014/02/25. 10.1016/j.ophtha.2014.01.002 . 24560564

[6] Chiu CJ, Chang ML, Zhang FF, Li T, Gensler G, Schleicher M, et al The relationship of major American dietary patterns to age-related macular degeneration. Am J Ophthalmol. 2014;158(1):118–27 e1. Epub 2014/05/06. 10.1016/j.ajo.2014.04.016 24792100 PMC4101985

[7] Schleicher M, Weikel K, Garber C, Taylor A. Diminishing risk for age-related macular degeneration with nutrition: a current view. Nutrients. 2013;5(7):2405–56. Epub 2013/07/04. 10.3390/nu5072405 23820727 PMC3738980

[8] Zampatti S, Ricci F, Cusumano A, Marsella LT, Novelli G, Giardina E. Review of nutrient actions on age-related macular degeneration. Nutrition research. 2014;34(2):95–105. Epub 2014/01/28. 10.1016/j.nutres.2013.10.011 . 24461310

[9] Delcourt C, Carriere I, Cristol JP, Lacroux A, Gerber M. Dietary fat and the risk of age-related maculopathy: the POLANUT study. European journal of clinical nutrition. 2007;61(11):1341–4. Epub 2007/02/15. 1602685 [pii] 10.1038/sj.ejcn.1602685 . 17299457

[10] Merle B, Delyfer MN, Korobelnik JF, Rougier MB, Colin J, Malet F, et al Dietary omega-3 fatty acids and the risk for age-related maculopathy: the Alienor Study. Invest Ophthalmol Vis Sci. 2011;52(8):6004–11. Epub 2011/06/28. 10.1167/iovs.11-7254 . 21705687

[11] Feart C, Samieri C, Alles B, Barberger-Gateau P. Potential benefits of adherence to the Mediterranean diet on cognitive health. The Proceedings of the Nutrition Society. 2013;72(1):140–52. Epub 2012/12/12. 10.1017/S0029665112002959 . 23228285

[12] Feart C, Samieri C, Rondeau V, Amieva H, Portet F, Dartigues JF, et al Adherence to a Mediterranean diet, cognitive decline, and risk of dementia. JAMA. 2009;302(6):638–48. Epub 2009/08/13. 302/6/638 [pii] 10.1001/jama.2009.1146 19671905 PMC2850376

[13] Pelletier A, Barul C, Feart C, Helmer C, Bernard C, Periot O, et al Mediterranean diet and preserved brain structural connectivity in older subjects. Alzheimer's & dementia: the journal of the Alzheimer's Association. 2015 Epub 2015/07/21. 10.1016/j.jalz.2015.06.1888 . 26190494

[14] Sofi F, Abbate R, Gensini GF, Casini A. Accruing evidence on benefits of adherence to the Mediterranean diet on health: an updated systematic review and meta-analysis. Am J Clin Nutr. 2010;92(5):1189–96. Epub 2010/09/03. 10.3945/ajcn.2010.29673 . 20810976

[15] Mares JA, Voland RP, Sondel SA, Millen AE, Larowe T, Moeller SM, et al Healthy lifestyles related to subsequent prevalence of age-related macular degeneration. Arch Ophthalmol. 2011;129(4):470–80. Epub 2010/12/15. archophthalmol.2010.314 [pii] 10.1001/archophthalmol.2010.314 21149749 PMC3075357

[16] Merle BM, Silver RE, Rosner B, Seddon JM. Adherence to a Mediterranean diet, genetic susceptibility, and progression to advanced macular degeneration: a prospective cohort study. Am J Clin Nutr. 2015;102(5):1196–206. Epub 2015/10/23. 10.3945/ajcn.115.111047 . 26490493 PMC4625588

[17] Cicerale S, Lucas L, Keast R. Biological activities of phenolic compounds present in virgin olive oil. International journal of molecular sciences. 2010;11(2):458–79. Epub 2010/04/14. 10.3390/ijms11020458 20386648 PMC2852848

[18] Martin-Pelaez S, Covas MI, Fito M, Kusar A, Pravst I. Health effects of olive oil polyphenols: recent advances and possibilities for the use of health claims. Molecular nutrition & food research. 2013;57(5):760–71. Epub 2013/03/02. 10.1002/mnfr.201200421 . 23450515

[19] Buckland G, Mayen AL, Agudo A, Travier N, Navarro C, Huerta JM, et al Olive oil intake and mortality within the Spanish population (EPIC-Spain). Am J Clin Nutr. 2012;96(1):142–9. Epub 2012/06/01. 10.3945/ajcn.111.024216 . 22648725

[20] Buckland G, Travier N, Barricarte A, Ardanaz E, Moreno-Iribas C, Sanchez MJ, et al Olive oil intake and CHD in the European Prospective Investigation into Cancer and Nutrition Spanish cohort. The British journal of nutrition. 2012;108(11):2075–82. Epub 2012/09/26. 10.1017/S000711451200298X . 23006416

[21] Guasch-Ferre M, Hu FB, Martinez-Gonzalez MA, Fito M, Bullo M, Estruch R, et al Olive oil intake and risk of cardiovascular disease and mortality in the PREDIMED Study. BMC medicine. 2014;12:78 Epub 2014/06/03. 10.1186/1741-7015-12-78 24886626 PMC4030221

[22] Martinez-Gonzalez MA, Dominguez LJ, Delgado-Rodriguez M. Olive oil consumption and risk of CHD and/or stroke: a meta-analysis of case-control, cohort and intervention studies. The British journal of nutrition. 2014;112(2):248–59. Epub 2014/04/30. 10.1017/S0007114514000713 . 24775425

[23] Samieri C, Feart C, Proust-Lima C, Peuchant E, Tzourio C, Stapf C, et al Olive oil consumption, plasma oleic acid, and stroke incidence: the Three-City Study. Neurology. 2011;77(5):418–25. Epub 2011/06/17. 10.1212/WNL.0b013e318220abeb . 21676914

[24] Zhu L, Liu Z, Feng Z, Hao J, Shen W, Li X, et al Hydroxytyrosol protects against oxidative damage by simultaneous activation of mitochondrial biogenesis and phase II detoxifying enzyme systems in retinal pigment epithelial cells. The Journal of nutritional biochemistry. 2010;21(11):1089–98. Epub 2010/02/13. 10.1016/j.jnutbio.2009.09.006 . 20149621

[25] Chong EW, Robman LD, Simpson JA, Hodge AM, Aung KZ, Dolphin TK, et al Fat consumption and its association with age-related macular degeneration. Arch Ophthalmol. 2009;127(5):674–80. Epub 2009/05/13. 127/5/674 [pii] 10.1001/archophthalmol.2009.60 . 19433719

[26] Delcourt C, Korobelnik JF, Barberger-Gateau P, Delyfer MN, Rougier MB, Le Goff M, et al Nutrition and age-related eye diseases: the Alienor (Antioxydants, Lipides Essentiels, Nutrition et maladies OculaiRes) Study. The journal of nutrition, health & aging. 2010;14(10):854–61. Epub 2010/12/03. 21125205 10.1007/s12603-010-0131-9 PMC3081304

[27] Vascular factors and risk of dementia: design of the Three-City Study and baseline characteristics of the study population. Neuroepidemiology. 2003;22(6):316–25. Epub 2003/11/06. . 14598854 10.1159/000072920

[28] Bird AC, Bressler NM, Bressler SB, Chisholm IH, Coscas G, Davis MD, et al An international classification and grading system for age-related maculopathy and age-related macular degeneration. The International ARM Epidemiological Study Group. Surv Ophthalmol. 1995;39(5):367–74. Epub 1995/03/01. . 7604360 10.1016/s0039-6257(05)80092-x

[29] Klein R, Klein BE, Knudtson MD, Wong TY, Cotch MF, Liu K, et al Prevalence of age-related macular degeneration in 4 racial/ethnic groups in the multi-ethnic study of atherosclerosis. Ophthalmology. 2006;113(3):373–80. Epub 2006/03/04. S0161-6420(05)01438-7 [pii] 10.1016/j.ophtha.2005.12.013 . 16513455

[30] Barberger-Gateau P, Raffaitin C, Letenneur L, Berr C, Tzourio C, Dartigues JF, et al Dietary patterns and risk of dementia: the Three-City cohort study. Neurology. 2007;69(20):1921–30. Epub 2007/11/14. 69/20/1921 [pii] 10.1212/01.wnl.0000278116.37320.52 . 17998483

[31] Larrieu S, Letenneur L, Berr C, Dartigues JF, Ritchie K, Alperovitch A, et al Sociodemographic differences in dietary habits in a population-based sample of elderly subjects: the 3C study. The journal of nutrition, health & aging. 2004;8(6):497–502. Epub 2004/11/16. . 15543423

[32] Feart C, Jutand MA, Larrieu S, Letenneur L, Delcourt C, Combe N, et al Energy, macronutrient and fatty acid intake of French elderly community dwellers and association with socio-demographic characteristics: data from the Bordeaux sample of the Three-City Study. The British journal of nutrition. 2007;98(5):1046–57. Epub 2007/05/30. S0007114507756520 [pii] 10.1017/S0007114507756520 . 17532868

[33] Centre National Hospitalier d'Information sur le Médicament (CNHIM) Theriaque website. available at: https://www.theriaque.org. accessed February 1, 2015.

[34] Feart C, Peuchant E, Letenneur L, Samieri C, Montagnier D, Fourrier-Reglat A, et al Plasma eicosapentaenoic acid is inversely associated with severity of depressive symptomatology in the elderly: data from the Bordeaux sample of the Three-City Study. Am J Clin Nutr. 2008;87(5):1156–62. Epub 2008/05/13. 87/5/1156 [pii]. . 18469234 10.1093/ajcn/87.5.1156

[35] Merle BM, Delyfer MN, Korobelnik JF, Rougier MB, Malet F, Feart C, et al High concentrations of plasma n3 fatty acids are associated with decreased risk for late age-related macular degeneration. The Journal of nutrition. 2013;143(4):505–11. Epub 2013/02/15. 10.3945/jn.112.171033 . 23406618

[36] Samieri C, Feart C, Letenneur L, Dartigues JF, Peres K, Auriacombe S, et al Low plasma eicosapentaenoic acid and depressive symptomatology are independent predictors of dementia risk. Am J Clin Nutr. 2008;88(3):714–21. Epub 2008/09/10. 88/3/714 [pii]. . 18779288 10.1093/ajcn/88.3.714

[37] Baird PN, Richardson AJ, Robman LD, Dimitrov PN, Tikellis G, McCarty CA, et al Apolipoprotein (APOE) gene is associated with progression of age-related macular degeneration (AMD). Human mutation. 2006;27(4):337–42. Epub 2006/02/03. 10.1002/humu.20288 . 16453339

[38] Scholl HP, Fleckenstein M, Charbel Issa P, Keilhauer C, Holz FG, Weber BH. An update on the genetics of age-related macular degeneration. Mol Vis. 2007;13:196–205. Epub 2007/03/01. v13/a23 [pii]. 17327825 PMC2610372

[39] Seddon JM, Francis PJ, George S, Schultz DW, Rosner B, Klein ML. Association of CFH Y402H and LOC387715 A69S with progression of age-related macular degeneration. JAMA. 2007;297(16):1793–800. Epub 2007/04/26. 297/16/1793 [pii] 10.1001/jama.297.16.1793 . 17456821

[40] Delcourt C, Delyfer MN, Rougier MB, Amouyel P, Colin J, Le Goff M, et al Associations of Complement Factor H and smoking with early age-related macular degeneration: the ALIENOR study. Invest Ophthalmol Vis Sci. 2011 Epub 2011/06/07. iovs.10-6235 [pii] 10.1167/iovs.10-6235 . 21642625

[41] Delcourt C, Delyfer MN, Rougier MB, Lambert JC, Amouyel P, Colin J, et al ARMS2 A69S polymorphism and the risk for age-related maculopathy: the ALIENOR study. Arch Ophthalmol. 2012;130(8):1077–8. Epub 2012/08/16. 10.1001/archophthalmol.2012.420 . 22893087

[42] Merle BM, Maubaret C, Korobelnik JF, Delyfer MN, Rougier MB, Lambert JC, et al Association of HDL-related loci with age-related macular degeneration and plasma lutein and zeaxanthin: the Alienor study. PLoS One. 2013;8(11):e79848 Epub 2013/11/14. 10.1371/journal.pone.0079848 24223199 PMC3819249

[43] Zeger SL, Liang KY, Albert PS. Models for longitudinal data: a generalized estimating equation approach. Biometrics. 1988;44(4):1049–60. Epub 1988/12/01. . 3233245

[44] Cougnard-Gregoire A, Delyfer MN, Korobelnik JF, Rougier MB, Le Goff M, Dartigues JF, et al Elevated high-density lipoprotein cholesterol and age-related macular degeneration: the Alienor study. PLoS One. 2014;9(3):e90973 Epub 2014/03/13. 10.1371/journal.pone.0090973 24608419 PMC3946623

[45] Reynolds R, Rosner B, Seddon JM. Dietary omega-3 fatty acids, other fat intake, genetic susceptibility, and progression to incident geographic atrophy. Ophthalmology. 2013;120(5):1020–8. Epub 2013/03/14. 10.1016/j.ophtha.2012.10.020 23481534 PMC3758110

[46] SanGiovanni JP, Chew EY, Clemons TE, Davis MD, Ferris FL 3rd, Gensler GR, et al The relationship of dietary lipid intake and age-related macular degeneration in a case-control study: AREDS Report No. 20. Arch Ophthalmol. 2007;125(5):671–9. Epub 2007/05/16. 125/5/671 [pii] 10.1001/archopht.125.5.671 . 17502507

[47] Seddon JM, Rosner B, Sperduto RD, Yannuzzi L, Haller JA, Blair NP, et al Dietary fat and risk for advanced age-related macular degeneration. Arch Ophthalmol. 2001;119(8):1191–9. Epub 2001/08/14. eeb00016 [pii]. . 11483088 10.1001/archopht.119.8.1191

[48] Seddon JM, Cote J, Rosner B. Progression of age-related macular degeneration: association with dietary fat, transunsaturated fat, nuts, and fish intake. Arch Ophthalmol. 2003;121(12):1728–37. Epub 2003/12/10. 10.1001/archopht.121.12.1728 121/12/1728 [pii]. . 14662593 PMC8443211

[49] Cho E, Hung S, Willett WC, Spiegelman D, Rimm EB, Seddon JM, et al Prospective study of dietary fat and the risk of age-related macular degeneration. Am J Clin Nutr. 2001;73(2):209–18. Epub 2001/02/07. . 11157315 10.1093/ajcn/73.2.209

[50] Parekh N, Voland RP, Moeller SM, Blodi BA, Ritenbaugh C, Chappell RJ, et al Association between dietary fat intake and age-related macular degeneration in the Carotenoids in Age-Related Eye Disease Study (CAREDS): an ancillary study of the Women's Health Initiative. Arch Ophthalmol. 2009;127(11):1483–93. Epub 2009/11/11. 127/11/1483 [pii] 10.1001/archophthalmol.2009.130 . 19901214 PMC3144752

[51] Smith W, Mitchell P, Leeder SR. Dietary fat and fish intake and age-related maculopathy. Arch Ophthalmol. 2000;118(3):401–4. Epub 2000/03/18. . 10721964 10.1001/archopht.118.3.401

[52] Parkinson L, Keast R. Oleocanthal, a phenolic derived from virgin olive oil: a review of the beneficial effects on inflammatory disease. International journal of molecular sciences. 2014;15(7):12323–34. Epub 2014/07/16. 10.3390/ijms150712323 25019344 PMC4139846

[53] Bulotta S, Celano M, Lepore SM, Montalcini T, Pujia A, Russo D. Beneficial effects of the olive oil phenolic components oleuropein and hydroxytyrosol: focus on protection against cardiovascular and metabolic diseases. Journal of translational medicine. 2014;12:219 Epub 2014/08/05. 10.1186/s12967-014-0219-9 25086598 PMC4237885

[54] EFSA, Panel, on, Dietetic, Products. Nutrition and allergies (NDA). Scientific opinion on the substantiation of health claims related to polyphenols in olive oil and protection of LDL particles from oxidative damage. EFSA Journal 2011;9:2033.; 2011.

[55] Castaner O, Covas MI, Khymenets O, Nyyssonen K, Konstantinidou V, Zunft HF, et al Protection of LDL from oxidation by olive oil polyphenols is associated with a downregulation of CD40-ligand expression and its downstream products in vivo in humans. Am J Clin Nutr. 2012;95(5):1238–44. Epub 2012/03/24. 10.3945/ajcn.111.029207 . 22440854

[56] Zerbib J, Delcourt C, Puche N, Querques G, Cohen SY, Sahel J, et al Risk factors for exudative age-related macular degeneration in a large French case-control study. Graefes Arch Clin Exp Ophthalmol. 2014;252(6):899–907. Epub 2013/12/24. 10.1007/s00417-013-2537-7 . 24362810

[57] Davis MD, Gangnon RE, Lee LY, Hubbard LD, Klein BE, Klein R, et al The Age-Related Eye Disease Study severity scale for age-related macular degeneration: AREDS Report No. 17. Arch Ophthalmol. 2005;123(11):1484–98. Epub 2005/11/16. 123/11/1484 [pii] 10.1001/archopht.123.11.1484 16286610 PMC1472813

[58] Klein R, Klein BE, Tomany SC, Meuer SM, Huang GH. Ten-year incidence and progression of age-related maculopathy: The Beaver Dam eye study. Ophthalmology. 2002;109(10):1767–79. Epub 2002/10/03. S0161-6420(02)01146-6 [pii]. . 12359593 10.1016/s0161-6420(02)01146-6

[59] van Leeuwen R, Klaver CC, Vingerling JR, Hofman A, de Jong PT. The risk and natural course of age-related maculopathy: follow-up at 6 1/2 years in the Rotterdam study. Arch Ophthalmol. 2003;121(4):519–26. Epub 2003/04/16. 10.1001/archopht.121.4.519 121/4/519 [pii]. . 12695249

[60] Wang JJ, Rochtchina E, Lee AJ, Chia EM, Smith W, Cumming RG, et al Ten-year incidence and progression of age-related maculopathy: the blue Mountains Eye Study. Ophthalmology. 2007;114(1):92–8. Epub 2007/01/03. S0161-6420(06)00998-5 [pii] 10.1016/j.ophtha.2006.07.017 . 17198852

[61] Augood CA, Vingerling JR, de Jong PT, Chakravarthy U, Seland J, Soubrane G, et al Prevalence of age-related maculopathy in older Europeans: the European Eye Study (EUREYE). Arch Ophthalmol. 2006;124(4):529–35. Epub 2006/04/12. 124/4/529 [pii] 10.1001/archopht.124.4.529 . 16606879

[62] Vingerling JR, Dielemans I, Hofman A, Grobbee DE, Hijmering M, Kramer CF, et al The prevalence of age-related maculopathy in the Rotterdam Study. Ophthalmology. 1995;102(2):205–10. Epub 1995/02/01. . 7862408 10.1016/s0161-6420(95)31034-2

[63] Friedman DS, O'Colmain BJ, Munoz B, Tomany SC, McCarty C, de Jong PT, et al Prevalence of age-related macular degeneration in the United States. Arch Ophthalmol. 2004;122(4):564–72. Epub 2004/04/14. 10.1001/archopht.122.4.564 122/4/564 [pii]. . 15078675

[64] Ollivier D, Artaud J, Pinatel C, Durbec JP, Guerere M. Triacylglycerol and fatty acid compositions of French virgin olive oils. Characterization by chemometrics. Journal of agricultural and food chemistry. 2003;51(19):5723–31. Epub 2003/09/04. 10.1021/jf034365p . 12952425

