# Supplementary material for: Olive Oil Consumption and Age-Related Macular Degeneration: The Alienor Study
Source: PLoS One. 2016 Jul 28;11(7):e0160240. doi: 10.1371/journal.pone.0160240 (PMC4965131; doi:10.1371/journal.pone.0160240)
Supplement: S1 Table — ALIENOR: Antioxydants, Lipides Essentiels, Nutrition et maladies OculaiRes; AMD: age-related macular degeneration; BMI: body mass index; HDL: High-density lipoprotein cholesterol; LDL: Low-density lipoprotein cholesterol; PUFA: polyunsaturated fatty acids; S.D.: standard deviation; * Values are means ± SDs or n (%).†Comparisons of characteristics with missing data status were examined with logistic regression analysis adjusted for age; ‖ Fasting blood glucose ≥ 7 mmol/L and/or nonfasting blood glucose ≥11.0 mmol/L and/or antidiabetic medication use; § Average systolic blood pressure ≥ 140 mmHg and/or average diastolic blood pressure ≥ 90 mmHg and/or antihypertensive medication use; {percentage of total fatty acids; # colza, walnut or soya oils; ** peanut, sunflower, grape or corn oils (DOCX) [file pone.0160240.s001.docx]

**S1 Table: comparison of the characteristics of subjects included with and without missing data (Alienor study 2006-2008, Bordeaux, France)**^*^

|  | **Participants with missing data**  **(N=309)** | **Participants**  **without missing data (N=654)** | **P-value**^†^ |
| --- | --- | --- | --- |
| **Age at baseline** | 73.5 ± 4.4 | 72.7 ± 4.3 | 0.007 |
| **Gender** |  |  |  |
| Men | 115 (37.2) | 253 (38.7) | 0.82 |
| women | 194 (62.8) | 401 (61.3) |  |
| **Education** (n=962) |  |  |  |
| None or Primary School | 95 (30.8) | 180 (27.5) | 0.77 |
| Secondary | 82 (26.6) | 181 (27.7) |  |
| High School or University | 131 (42.5) | 293 (44.8) |  |
| **Monthly Income (in euros)**(n=957) |  |  |  |
| <1500 | 116 (38.3) | 239 (36.5) | 0.13 |
| [1500-2250[ | 72 (23.8) | 178 (27.2) |  |
| ≥ 2250 | 91 (30.0) | 208 (31.8) |  |
| Refused to answer | 24 (7.9) | 29 (4.4) |  |
| **Marital status** |  |  |  |
| Married | 164 (53.1) | 415 (63.5) | 0.008 |
| Divorced, widowed or single | 145 (46.9) | 239 (36.5) |  |
| **Smoking (pack-years)** (n=951) |  |  | 0.58 |
| None | 191(64.3) | 423 (64.7) |  |
| <20 | 59 (19.9) | 114 (17.4) |  |
| ≥20 | 47 (15.8) | 117 (17.9) |  |
| **Alcohol use** (number of glasses per week) (n=956) | 9.9 ± 11.1 | 10.7 ± 12.2 | 0.39 |
| **Physical activity** |  |  |  |
| None | 142 (46.0) | 363 (55.5) | <0.0001 |
| Medium | 51 (16.5) | 139 (21.3) |  |
| High | 19 (6.2) | 71 (10.9) |  |
| Not answered | 97 (31.4) | 81 (12.4) |  |

**S1 Table (cont.): comparison of the characteristics of subjects included with and without missing data (Alienor study 2006-2008, Bordeaux, France)**^*^

|  | | **Participants** | **Participants** | **P-value**^†^ |
| --- | --- | --- | --- | --- |
|  | | **with missing** | **without missing** |  |
|  | | **data** | **data** |  |
|  | | **(N=309)** | **(N=654)** |  |
| **AMD** | |  |  |  |
| No | | 151 (67.1) | 429 (65.6) | 0.84 |
| Early | | 61 (27.1) | 189 (28.9) |  |
| Late | | 13 (5.8) | 36 (5.5) |  |
| **History of cardiovascular disease** | |  |  |  |
| No | | 282 (91.3) | 601 (91.9) | 0.74 |
| Yes | | 27 (8.7) | 53 (8.1) |  |
| **Diabetes**^‖^ (n=892) | |  |  |  |
| No | | 210 (88.2) | 606 (92.7) | 0.04 |
| Yes | | 28 (11.8) | 48 (7.3) |  |
| **Hypertension**^§^ | |  |  |  |
| No | | 66 (21.4) | 168 (25.7) | 0.25 |
| Yes | | 243 (78.6) | 486 (74.3) |  |
| **BMI, means (SD)** (n=955) | | 26.5 (4.2) | 26.3 (3.8) | 0.53 |
| **Plasma total cholesterol (mmol/L)**(n=902) | | 5.9 ± 1.0 | 5.8 ± 0.9 | 0.20 |
| **Plasma LDL-cholesterol (mmol/L)** (n=900) | | 3.7 ± 0.9 | 3.6 ± 0.8 | 0.16 |
| **Plasma HDL-cholesterol (mmol/L)** (n=901) | | 1.6 ± 0.4 | 1.6 ± 0.4 | 0.18 |
| **Plasma triglycerides (mmol/L)**(n=901) | 1.3 ± 0.6 | | 1.2 ± 0.6 | 0.02 |
| **Plasma Oleic acid**^{^  (n=777) | 20.7 ± 3.2 | | 20.7 ± 3.3 | 0.89 |
| **Plasma n-3 PUFAs**^{^  (n=777) | 4.4 ± 1.4 | | 4.5 ± 1.3 | 0.85 |
| **Plasma n-6 PUFAs**^{^  (n=777) | 32.8 ± 5.1 | | 33.0 ± 4.9 | 0.88 |
| **Plasma saturated fatty acids**^{^ (n=777) | 39.7 ± 4.8 | | 39.6 ± 5.5 | 0.99 |
| ***CFH* rs1061170** (n=878) |  | |  |  |
| TT (low AMD risk) | 108 (44.3) | | 291 (45.9) | 0.74 |
| TC | 105 (43.0) | | 273 (43.1) |  |
| CC (high AMD risk) | 31 (12.7) | | 70 (11.0) |  |
| ***ARMS2* rs10490924** (n=583) |  | |  |  |
| GG (low AMD risk) | 151 (67.7) | | 373 (64.0) | 0.27 |
| GT | 68 (30.5) | | 186 (31.9) |  |
| TT (high AMD risk) | 4 (1.8) | | 24 (4.1) |  |
| ***LPL* rs12678919** (n=782) |  | |  |  |
| A A (low AMD risk) | 165 (76.7) | | 416 (73.4) | 0.54 |
| A G | 47 (21.9) | | 139 (24.5) |  |
| G G (high AMD risk) | 3 (1.4) | | 12 (2.1) |  |
| ***LIPC* rs493258** (n=806) |  | |  |  |
| C C (high AMD risk) | 71 (31.8) | | 166 (28.5) | 0.53 |
| T C | 107 (48.0) | | 282 (48.4) |  |
| T T (low AMD risk) | 45 (20.2) | | 135 (23.2) |  |

**S1 Table (cont.): comparison of the characteristics of subjects included with and without missing data (Alienor study 2006-2008, Bordeaux, France)^*^**

|  | **Participants with** | **Participants** | **P-value**^†^ |
| --- | --- | --- | --- |
|  | **missing data** | **without** |  |
|  |  | **missing data** |  |
|  | **(N=309)** | **(N=654)** |  |
| **Regular consumption of** |  |  |  |
| Fish (≥ once a week) (n=962) |  |  |  |
| No | 33 (10.7) | 57 (8.7) | 0.41 |
| Yes | 275 (89.3) | 597 (91.3) |  |
| Meat (≥ twice a week) (n=962) |  |  |  |
| No | 17 (5.5) | 31 (4.7) | 0.60 |
| Yes | 291 (94.5) | 623 (95.3) |  |
| Raw vegetables (≥ twice a week) (n=962) |  |  |  |
| No | 42 (13.6) | 56 (8.6) | 0.03 |
| Yes | 266 (86.4) | 598 (91.4) |  |
| Raw fruits (≥ 4 times a week) (n=962) |  |  |  |
| No | 39 (12.62) | 94 (14.4) | 0.52 |
| Yes | 270 (87.4) | 560 (85.6) |  |
| Cooked fruits and vegetables (≥ 4 times a week) (n=962) |  |  |  |
| No | 33 (10.7) | 72 (11.0) | 0.98 |
| Yes | 275 (89.3) | 582 (89.0) |  |
| Legumes (≥ once a week) |  |  |  |
| No | 38 (12.3) | 56 (8.6) | 0.06 |
| Yes | 271 (87.7) | 598 (91.4) |  |
| Dairy products (once a day) |  |  |  |
| No | 24 (7.8) | 35 (5.4) | 0.17 |
| Yes | 285 (92.2) | 619 (94.6) |  |
| Eggs (once a week) (n=961) |  |  |  |
| No | 70 (22.8) | 123 (18.8) | 0.12 |
| Yes | 237 (77.2) | 531 (81.2) |  |
| **Preferred fats use** |  |  |  |
| Olive oil (n=958) |  |  |  |
| No | 105 (34.5) | 175 (26.8) | 0.02 |
| Yes | 199 (65.5) | 479 (73.2) |  |
| n-3 rich oils^#^ (n=958) |  |  |  |
| No | 288 (94.7) | 607 (92.8) | 0.28 |
| Yes | 16 (5.3) | 47 (7.2) |  |
| n-6 rich oils^**^ (n=958) |  |  |  |
| No | 93 (30.6) | 206 (31.5) | 0.79 |
| Yes | 211 (69.4) | 448 (68.5) |  |
| Mixed oils (n=958) |  |  |  |
| No | 261 (85.9) | 526 (80.4) | 0.06 |
| Yes | 43 (14.1) | 128 (19.6) |  |
| Butter (n=958) |  |  |  |
| No | 124 (40.8) | 270 (41.3) | 0.93 |
| Yes | 180 (59.2) | 384 (58.7) |  |
| Margarine (n=958) |  |  |  |
| No | 261 (85.9) | 526 (80.4) | 0.001 |
| Yes | 43 (14.1) | 128 (19.6) |  |

ALIENOR: Antioxydants, Lipides Essentiels, Nutrition et maladies OculaiRes; S.D.: standard deviation; AMD: age-related macular degeneration; BMI: body mass index; HDL: High-density lipoprotein cholesterol; LDL: Low-density lipoprotein cholesterol; PUFAs: polyunsaturated fatty acids;

^*^ Values are means ± SDs or n (%).

^†^ Comparisons of characteristics with missing data status were examined with logistic regression analysis adjusted for age;

^‖^ Fasting blood glucose ≥ 7 mmol/L and/or nonfasting blood glucose ≥11.0 mmol/L and/or antidiabetic medication use;

^§^ Average systolic blood pressure ≥ 140 mmHg and/or average diastolic blood pressure ≥ 90 mmHg and/or antihypertensive medication use;

^{^ percentage of total fatty acids;

^#^ colza, walnut or soya oils;

^**^ peanut, sunflower, grape or corn oil
