# Supplementary material for: Olive Oil Consumption and Age-Related Macular Degeneration: The Alienor Study
Source: PLoS One. 2016 Jul 28;11(7):e0160240. doi: 10.1371/journal.pone.0160240 (PMC4965131; doi:10.1371/journal.pone.0160240)
Supplement: S2 Table — ALIENOR: Antioxydants, Lipides Essentiels, Nutrition et maladies OculaiRes; S.D.: standard deviation; AMD: age-related macular degeneration; BMI: body mass index; HDL: High-density lipoprotein cholesterol; LDL: Low-density lipoprotein cholesterol; PUFAs: polyunsaturated fatty acids; * Values are means ± SDs or %. † Chi-square or fisher exact test for categorical variables and Student test for continuous variables; § Average systolic blood pressure ≥ 140 mmHg and/or average diastolic blood pressure ≥ 90 mmHg and/or antihypertensive medication use; ‖ Fasting blood glucose ≥ 7 mmol/L and/or nonfasting blood glucose ≥11.0 mmol/L and/or antidiabetic medication use; {percentage of total fatty acids; # colza, walnut or soya oils; ** peanut, sunflower, grape or corn oils. (DOCX) [file pone.0160240.s002.docx]

**S2 Table: comparison of the characteristics of subjects with n-3, n-6 rich oils and mixed oil use (Alienor study 2006-2008, Bordeaux, France)**

|  | **n-3 rich oils** | | | **n-6 rich oils** | | | **Mixed oils** | | |
| --- | --- | --- | --- | --- | --- | --- | --- | --- | --- |
|  | **Non user** | **Regular** | **P**^†^ | **Non user** | **Regular user** | **P**^†^ | **Non user** | **Regular user** | **P**^†^ |
|  | **(n=607)** | **user (n=47)** |  | **(n=206)** | **(n=448)** |  | **(n=526)** | **(n=128)** |  |
| **Age at baseline** | 72.7±4.3 | 72.9±4.0 | 0.79 | 72.9±4.3 | 72.7±4.2 | 0.53 | 72.8±4.4 | 72.4±4.2 | 0.34 |
| **Gender, women** | 61.6 | 57.5 | 0.57 | 65.5 | 59.4 | 0.13 | 61.2 | 61.7 | 0.92 |
| **Education** |  |  |  |  |  |  |  |  |  |
| None or Primary School | 26.9 | 36.2 | 0.38 | 19.9 | 31.0 | 0.003 | 29.5 | 19.5 | 0.06 |
| Secondary | 27.8 | 25.5 |  | 26.7 | 28.1 |  | 27.6 | 28.1 |  |
| High School or University | 45.3 | 38.3 |  | 53.4 | 40.9 |  | 43.0 | 52.3 |  |
| **Monthly Income (in euros)** |  |  |  |  |  |  |  |  |  |
| <1500 | 36.6 | 36.2 | 0.99 | 34.0 | 37.7 | 0.16 | 38.8 | 27.3 | 0.02 |
| [1500-2250[ | 27.4 | 25.5 |  | 23.8 | 28.8 |  | 27.8 | 25.0 |  |
| ≥ 2250 | 31.6 | 34.0 |  | 36.4 | 29.7 |  | 29.5 | 41.4 |  |
| Refused to answer | 4.5 | 4.3 |  | 5.8 | 3.8 |  | 4.0 | 6.3 |  |
| **Marital status,** Divorced, widowed or single | 37.4 | 25.5 | 0.10 | 40.8 | 34.6 | 0.13 | 37.6 | 32.0 | 0.24 |
| **Smoking (pack-years)** |  |  |  |  |  |  |  |  |  |
| None | 64.4 | 68.1 | 0.68 | 67.0 | 63.6 | 0.44 | 62.4 | 74.2 | 0.03 |
| <20 | 17.8 | 12.8 |  | 18.0 | 17.2 |  | 19.0 | 10.9 |  |
| ≥20 | 17.8 | 19.2 |  | 15.1 | 19.2 |  | 18.6 | 14.8 |  |
| **Alcohol use** (number of glasses per week) | 10.4±12.1 | 14.9±12.9 | 0.01 | 10.5±13.0 | 10.8±11.9 | 0.75 | 10.9±12.5 | 9.9±11.2 | 0.38 |
| **Physical activity** |  |  |  |  |  |  |  |  |  |
| None | 55.4 | 57.5 | 0.66 | 53.4 | 56.5 | 0.75 | 56.5 | 51.6 | 0.32 |
| Medium | 21.6 | 17.0 |  | 22.3 | 20.8 |  | 21.3 | 21.1 |  |
| High | 11.0 | 8.5 |  | 10.2 | 11.2 |  | 11.0 | 10.2 |  |
| Not answered | 12.0 | 17.0 |  | 14.1 | 11.6 |  | 11.2 | 17.20 |  |

**S2 Table (cont.): comparison of the characteristics of subjects with n-3, n-6 rich oils and mixed oil use (Alienor study 2006-2008, Bordeaux, France)**

|  | **n-3 rich oils** | | | **n-6 rich oils** | | | **Mixed oils** | | |
| --- | --- | --- | --- | --- | --- | --- | --- | --- | --- |
|  | **Non user** | **Regular** | **P**^†^ | **Non user** | **Regular user** | **P**^†^ | **Non user** | **Regular** | **P**^†^ |
|  |  | **user** |  |  |  |  |  | **user** |  |
|  | **(n=607)** | **(n=47)** |  | **(n=206)** | **(n=448)** |  | **(n=526)** | **(n=128)** |  |
| **Hypertension**^§^ | 73.8 | 80.9 | 0.29 | 73.3 | 74.8 | 0.69 | 73. | 76.6 | 0.52 |
| **SBP (mmHg)** | 143.0±20.0 | 143.7±22.6 | 0.82 | 140.7±20.6 | 144.1±20.0 | 0.04 | 143.6±20.2 | 140.8±20.0 | 0.16 |
| **DBP (mmHg)** | 81.3±10.5 | 82.3±11.0 | 0.51 | 79.9±10.7 | 82.1±10.4 | 0.01 | 81.4±10.4 | 81.2±11.2 | 0.87 |
| **Antihypertensive therapy** | 50.1 | 57.5 | 0.33 | 48.5 | 51.6 | 0.47 | 50.6 | 50.8 | 0.97 |
| **Diabetes**^‖^ | 7.6 | 4.3 | 0.40 | 7.3 | 7.4 | 0.97 | 7.2 | 7.8 | 0.82 |
| **Hypercholesterolemia** | 51.7 | 59.6 | 0.30 | 51.0 | 52.9 | 0.65 | 51.5 | 55.5 | 0.42 |
| **History of cardiovascular disease** | 7.4 | 17.0 | 0.02 | 7.3 | 8.5 | 0.60 | 7.6 | 10.2 | 0.34 |
| **BMI** | 26.3±3.8 | 27.1±3.3 | 0.13 | 26.0±3.8 | 26.4±3.8 | 0.21 | 26.3±3.8 | 26.3±3.8 | 0.95 |
| **Plasma total cholesterol (mmol/L)** | 5.8±1.0 | 5.7±0.8 | 0.51 | 5.7±0.9 | 5.8±1.0 | 0.38 | 5.8±1.0 | 5.6±0.9 | 0.04 |
| **Plasma LDL-cholesterol (mmol/L)** | 3.6±0.8 | 3.5±0.8 | 0.43 | 3.6±0.8 | 3.6±0.8 | 0.34 | 1.6±0.4 | 1.6±0.4 | 0.57 |
| **Plasma HDL-cholesterol (mmol/L)** | 1.6±0.4 | 1.6±0.3 | 0.91 | 1.6±0.4 | 1.6±0.4 | 0.72 | 3.6±0.9 | 3.4±0.8 | 0.01 |
| **Plasma triglycerides (mmol/L)** | 1.2±0.6 | 1.2±0.5 | 0.96 | 1.2±0.6 | 1.2±0.6 | 0.51 | 1.2±0.6 | 1.3±0.6 | 0.09 |
| **Plasma Oleic acid**^{^ | 20.7±3.3 | 20.1±2.7 | 0.20 | 21.4±3.2 | 20.3±3.3 | <0.0001 | 20.6±3.3 | 21.0±3.1 | 0.22 |
| **Plasma n-3 PUFAs**^{^ | 4.5±1.3 | 4.6±1.4 | 0.61 | 4.7±1.3 | 4.4±1.2 | 0.003 | 4.4±1.3 | 4.5±1.2 | 0.43 |
| **Plasma n-6 PUFAs**^{^ | 33.0±4.9 | 33.3±5.6 | 0.66 | 32.0±4.3 | 33.4±5.1 | 0.0001 | 33.1±5.1 | 32.5±4.3 | 0.19 |
| **Plasma saturated fatty acids**^{^ | 39.6±5.4 | 39.9±6.8 | 0.81 | 39.6±5.1 | 39.7±5.7 | 0.85 | 39.7±5.5 | 39.6±5.4 | 0.95 |

**S2 Table (cont.): comparison of the characteristics of subjects with n-3, n-6 rich oils and mixed oil use (Alienor study 2006-2008, Bordeaux, France)**^*^

|  | **n-3 rich oils** | | | **n-6 rich oils** | | | **Mixed oils** | | |
| --- | --- | --- | --- | --- | --- | --- | --- | --- | --- |
|  | **Non user** | **Regular user** | **P**^†^ | **Non user** | **Regular user** | **P**^†^ | **Non user** | **Regular user** | **P**^†^ |
|  | **(n=607)** | **(n=47)** |  | **(n=206)** | **(n=448)** |  | **(n=526)** | **(n=128)** |  |
| ***CFH* rs1061170** (n=878) |  |  |  |  |  |  |  |  |  |
| TT (low AMD risk) | 45.3 | 53.2 | 0.56 | 42.7 | 47.4 | 0.06 | 44.7 | 50.8 | 0.42 |
| TC | 43.4 | 38.3 |  | 49.3 | 40.2 |  | 43.7 | 40.3 |  |
| CC (high AMD risk) | 11.2 | 8.5 |  | 8.0 | 12.4 |  | 11.6 | 8.9 |  |
| ***ARMS2* rs10490924** (n=583) |  |  |  |  |  |  |  |  |  |
| GG (low AMD risk) | 64.0 | 63.6 | 0.79 | 64.5 | 63.7 | 0.11 | 63.3 | 67.0 | 0.45 |
| GT | 31.7 | 34.1 |  | 29.0 | 33.3 |  | 32.9 | 27.5 |  |
| TT (high AMD risk) | 4.3 | 2.3 |  | 6.5 | 3.0 |  | 3.8 | 5.5 |  |
| ***LPL* rs12678919** (n=782) |  |  |  |  |  |  |  |  |  |
| A A (low AMD risk) | 73.0 | 78.6 | 0.72 | 70.3 | 74.8 | 0.29 | 75.2 | 65.7 | 0.04 |
| A G | 24.8 | 21.4 |  | 26.4 | 23.6 |  | 23.3 | 29.6 |  |
| G G (high AMD risk) | 2.3 | 0.0 |  | 3.3 | 1.6 |  | 1.5 | 4.6 |  |
| ***LIPC* rs493258** (n=806) |  |  |  |  |  |  |  |  |  |
| C C (high AMD risk) | 28.2 | 31.8 | 0.77 | 26.9 | 29.2 | 0.45 | 28.5 | 28.4 | 0.37 |
| T C | 48.8 | 43.2 |  | 52.2 | 46.6 |  | 47.3 | 53.2 |  |
| T T (low AMD risk) | 23.0 | 25.0 |  | 21.0 | 24.2 |  | 24.3 | 18.4 |  |
| **Regular consumption of** |  |  |  |  |  |  |  |  |  |
| Fish (≥ once a week) | 91.1 | 93.6 | 0.56 | 89.8 | 92.0 | 0.36 | 91.4 | 90.6 | 0.77 |
| Meat (≥ twice a week) | 95.1 | 97.9 | 0.38 | 94.7 | 95.5 | 0.63 | 95.1 | 96.1 | 0.62 |
| Raw vegetables (≥ twice a week) | 91.4 | 91.5 | 0.99 | 92.2 | 91.1 | 0.62 | 90.5 | 95.3 | 0.08 |
| Raw fruits (≥ 4 times a week) | 86.2 | 78.7 | 0.16 | 87.4 | 84.8 | 0.39 | 84.2 | 91.4 | 0.04 |
| Cooked fruits and vegetables (≥ 4 times a week) | 88.5 | 95.7 | 0.12 | 90.8 | 88.2 | 0.32 | 88.2 | 92.2 | 0.20 |
| Legumes (≥ once a week) | 91.4 | 91.5 | 0.99 | 89.3 | 92.4 | 0.19 | 90.9 | 93.8 | 0.30 |
| Dairy products (once a day) | 94.7 | 93.6 | 0.74 | 95.6 | 94.2 | 0.45 | 94.1 | 96.9 | 0.21 |
| Eggs (once a week) (n=961) | 80.2 | 93.6 | 0.02 | 78.6 | 82.4 | 0.26 | 81.0 | 82.0 | 0.79 |

ALIENOR: Antioxydants, Lipides Essentiels, Nutrition et maladies OculaiRes; S.D.: standard deviation; AMD: age-related macular degeneration; BMI: body mass index; HDL: High-density lipoprotein cholesterol; LDL: Low-density lipoprotein cholesterol; PUFAs: polyunsaturated fatty acids;

^*^ Values are means ± SDs or %.

^†^ Chi-square or fisher exact test for categorical variables and Student test for continuous variables;

^§^ Average systolic blood pressure ≥ 140 mmHg and/or average diastolic blood pressure ≥ 90 mmHg and/or antihypertensive medication use;

^‖^ Fasting blood glucose ≥ 7 mmol/L and/or nonfasting blood glucose ≥11.0 mmol/L and/or antidiabetic medication use;

^{^ percentage of total fatty acids;

^#^ colza, walnut or soya oils;

^**^ peanut, sunflower, grape or corn oils
