# Supplementary material for: Olive Oil Consumption and Age-Related Macular Degeneration: The Alienor Study
Source: PLoS One. 2016 Jul 28;11(7):e0160240. doi: 10.1371/journal.pone.0160240 (PMC4965131; doi:10.1371/journal.pone.0160240)
Supplement: S3 Table — ALIENOR: Antioxydants, Lipides Essentiels, Nutrition et maladies OculaiRes; S.D.: standard deviation; AMD: age-related macular degeneration; BMI: body mass index; HDL: High-density lipoprotein cholesterol; LDL: Low-density lipoprotein cholesterol; PUFAs: polyunsaturated fatty acids; * Values are means ± SDs or %. † Chi-square or fisher exact test for categorical variables and Student test for continuous variables;§Average systolic blood pressure ≥ 140 mmHg and/or average diastolic blood pressure ≥ 90 mmHg and/or antihypertensive medication use; ‖ Fasting blood glucose ≥ 7 mmol/L and/or nonfasting blood glucose ≥11.0 mmol/L and/or antidiabetic medication use; {percentage of total fatty acids; # colza, walnut or soya oils; ** peanut, sunflower, grape or corn oils. (DOCX) [file pone.0160240.s003.docx]

**S3 Table: comparison of the characteristics of subjects with butter or margarine use (Alienor study 2006-2008, Bordeaux, France)**^*^

|  | **Butter** | | | **Margarine** | | |
| --- | --- | --- | --- | --- | --- | --- |
|  | **Non user** | **Regular user** | **P**^†^ | **Non user** | **Regular user** | **P**^†^ |
|  | **(n=270)** | **(n=384)** |  | **(n=452)** | **(n=202)** |  |
| **Age at baseline** | 72.7±4.0 | 72.8±4.5 | 0.78 | 72.6±4.4 | 73.0±4.1 | 0.36 |
| **Gender, women** | 61.1 | 61.5 | 0.93 | 62.6 | 58.4 | 0.31 |
| **Education** |  |  |  |  |  |  |
| None or Primary School | 28.5 | 26.8 | 0.13 | 29.2 | 23.8 | 0.36 |
| Secondary | 25.3 | 25.3 |  | 27.0 | 29.2 |  |
| High School or University | 47.9 | 47.9 |  | 43.8 | 47.0 |  |
| **Monthly Income (in euros)** |  |  |  |  |  |  |
| <1500 | 36.7 | 36.5 | 0.08 | 38.1 | 33.2 | 0.13 |
| [1500-2250[ | 29.3 | 25.8 |  | 27.0 | 27.7 |  |
| ≥ 2250 | 27.8 | 34.6 |  | 29.7 | 36.6 |  |
| Refused to answer | 6.3 | 3.1 |  | 5.3 | 2.5 |  |
| **Marital status** |  |  |  |  |  |  |
| Divorced, widowed or single | 38.9 | 34.9 | 0.30 | 38.7 | 31.7 | 0.08 |
| **Smoking (pack-years)** |  |  |  |  |  |  |
| None | 66.3 | 63.5 | 0.74 | 63.9 | 66.3 | 0.52 |
| <20 | 17.0 | 17.7 |  | 17.0 | 18.3 |  |
| ≥20 | 16.7 | 18.8 |  | 19.0 | 15.4 |  |
| **Alcohol use** (number of glasses per week) | 10.2±12.7 | 11.1±11.9 | 0.36 | 11.3±12.0 | 10.5±12.3 | 0.43 |
| **Physical activity** |  |  |  |  |  |  |
| None | 59.6 | 52.6 | 0.30 | 54.7 | 57.4 | 0.04 |
| Medium | 18.9 | 22.9 |  | 20.6 | 22.8 |  |
| High | 10.7 | 10.9 |  | 10.0 | 12.9 |  |
| Not answered | 10.7 | 13.5 |  | 14.8 | 6.9 |  |

**S3 Table (cont.): comparison of the characteristics of subjects with butter or margarine use (Alienor study 2006-2008, Bordeaux, France)**

|  | **Butter** | | | **Margarine** | | |
| --- | --- | --- | --- | --- | --- | --- |
|  | **Non user** | **Regular user** | **P**^†^ | **Non user** | **Regular user** | **P**^†^ |
|  | **(n=270)** | **(n=384)** |  | **(n=452)** | **(n=202)** |  |
| **Hypertension**^§^ | 74.1 | 74.5 | 0.91 | 75.2 | 72.3 | 0.43 |
| **SBP (mmHg)** | 142.7±19.6 | 143.3±20.7 | 0.68 | 143.0±19.8 | 143.2±21.2 | 0.90 |
| **DBP (mmHg)** | 80.2±10.8 | 82.2±10.3 | 0.02 | 81.7±10.5 | 80.7±10.7 | 0.25 |
| **Antihypertensive therapy** | 51.1 | 50.3 | 0.83 | 51.6 | 48.5 | 0.47 |
| **Diabetes**^‖^ | 7.8 | 7.0 | 0.72 | 8.0 | 5.9 | 0.36 |
| **Hypercholesterolemia** | 52.6 | 52.1 | 0.90 | 53.5 | 49.5 | 0.34 |
| **History of cardiovascular disease** | 8.2 | 8.1 | 0.97 | 8.4 | 7.4 | 0.67 |
| **BMI** | 26.7±3.9 | 26.0±3.8 | 0.02 | 26.5±3.9 | 26.0±3.6 | 0.11 |
| **Plasma total cholesterol (mmol/L)** | 5.7±0.9 | 5.8±1.0 | 0.72 | 5.8±1.00 | 5.8±0.9 | 0.71 |
| **Plasma LDL-cholesterol (mmol/L)** | 3.6±0.8 | 3.6±0.9 | 0.80 | 3.6±0.9 | 3.6±0.8 | 0.82 |
| **Plasma HDL-cholesterol (mmol/L)** | 1.6±0.4 | 1.6±0.4 | 0.07 | 1.6±0.4 | 1.6±0.4 | 0.40 |
| **Plasma triglycerides (mmol/L)** | 1.2±0.6 | 1.2±0.6 | 0.54 | 1.2±0.6 | 1.2±0.6 | 0.51 |
| **Plasma Oleic acid**^{^ | 20.7±3.2 | 20.6±3.4 | 0.79 | 20.8±3.5 | 20.4±2.8 | 0.14 |
| **Plasma n-3 PUFAs**^{^ | 4.4±1.2 | 4.6±1.4 | 0.11 | 4.4±1.3 | 4.5±1.3 | 0.37 |
| **Plasma n-6 PUFAs**^{^ | 32.7±5.3 | 33.1±4.7 | 0.31 | 32.6±4.9 | 33.8±5.0 | 0.004 |
| **Plasma saturated fatty acids**^{^ | 39.7±6.2 | 39.6±4.9 | 0.70 | 39.9±5.6 | 39.1±5.3 | 0.09 |
| ***CFH* rs1061170** |  |  |  |  |  |  |
| TT (low AMD risk) | 48.3 | 44.2 | 0.48 | 46.1 | 45.5 | 0.95 |
| TC | 40.2 | 45.0 |  | 43.1 | 42.9 |  |
| CC (high AMD risk) | 11.5 | 10.7 |  | 10.8 | 11.6 |  |

**S3 Table (cont.): comparison of the characteristics of subjects with butter or margarine use (Alienor study 2006-2008, Bordeaux, France)**^*^

|  | **Butter** | | | **Margarine** | | |
| --- | --- | --- | --- | --- | --- | --- |
|  | **Non user** | **Regular user** | **P**^†^ | **Non user** | **Regular user** | **P**^†^ |
|  | **(n=270)** | **(n=384)** |  | **(n=452)** | **(n=202)** |  |
| ***ARMS2* rs10490924** |  |  |  |  |  |  |
| GG (low AMD risk) | 60.5 | 66.5 | 0.30 | 64.0 | 63.8 | 0.99 |
| GT | 35.4 | 29.4 |  | 31.8 | 32.2 |  |
| TT (high AMD risk) | 4.1 | 4.1 |  | 4.2 | 4.0 |  |
| ***LPL* rs12678919** |  |  |  |  |  |  |
| A A (low AMD risk) | 74.5 | 72.6 | 0.06 | 73.9 | 72.3 | 0.69 |
| A G | 25.1 | 24.1 |  | 24.4 | 24.9 |  |
| G G (high AMD risk) | 0.4 | 3.3 |  | 1.8 | 2.9 |  |
| ***LIPC* rs493258** |  |  |  |  |  |  |
| C C (high AMD risk) | 28.4 | 28.5 | 0.66 | 27.8 | 29.9 | 0.47 |
| T C | 50.2 | 47.1 |  | 50.0 | 44.6 |  |
| T T (low AMD risk) | 21.4 | 24.4 |  | 22.2 | 25.4 |  |
| **Regular consumption of** |  |  |  |  |  |  |
| Fish (≥ once a week) | 92.2 | 90.6 | 0.48 | 89.8 | 94.6 | 0.05 |
| Meat (≥ twice a week) | 95.2 | 95.3 | 0.94 | 94.5 | 97.0 | 0.16 |
| Raw vegetables (≥ twice a week) | 92.2 | 90.9 | 0.55 | 90.7 | 93.1 | 0.32 |
| Raw fruits (≥ 4 times a week) | 85.6 | 85.7 | 0.97 | 85.2 | 86.6 | 0.62 |
| Cooked fruits and vegetables (≥ 4 times a week) | 89.3 | 88.8 | 0.85 | 87.2 | 93.1 | 0.03 |
| Legumes (≥ once a week) | 91.9 | 91.2 | 0.75 | 90.3 | 94.1 | 0.11 |
| Dairy products (once a day) | 93.0 | 95.8 | 0.11 | 94.3 | 95.5 | 0.50 |
| Eggs (once a week) | 76.3 | 84.6 | 0.007 | 81.4 | 80.7 | 0.83 |

ALIENOR: Antioxydants, Lipides Essentiels, Nutrition et maladies OculaiRes; S.D.: standard deviation; AMD: age-related macular degeneration; BMI: body mass index; HDL: High-density lipoprotein cholesterol; LDL: Low-density lipoprotein cholesterol; PUFAs: polyunsaturated fatty acids;

^*^ Values are means ± SDs or %.

^†^ Chi-square or fisher exact test for categorical variables and Student test for continuous variables;

^§^ Average systolic blood pressure ≥ 140 mmHg and/or average diastolic blood pressure ≥ 90 mmHg and/or antihypertensive medication use;

^‖^ Fasting blood glucose ≥ 7 mmol/L and/or nonfasting blood glucose ≥11.0 mmol/L and/or antidiabetic medication use;

^{^ percentage of total fatty acids;

^#^ colza, walnut or soya oils;

^**^ peanut, sunflower, grape or corn oils
